# Comparative analysis of predictors of child mortality in Ethiopia via frequentist and Bayesian approaches

**DOI:** 10.1038/s41598-025-02059-y

**Published:** 2025-08-27

**Authors:** Aragaw Eshetie Aguade, Ding-Geng Chen

**Affiliations:** 1 Department of Statistics, College of Natural and Computational Sciences, University of Gondar, Gondar, Ethiopia; 2 Department of Statistics, University of Pretoria, Pretoria, South Africa; 3 College of Health Solutions, Arizona State University, Phoenix, AZ 85004 USA

**Keywords:** Ethiopia, Under-five mortality, Frequentist, Bayesian, Prevalence, DHS, Computational biology and bioinformatics, Diseases, Health care, Health occupations, Mathematics and computing

## Abstract

The child mortality rate is a leading factor in the well-being and development of a nation. It measures the quality of life for a given population. This study aimed to determine the effects of under-five child mortality in Ethiopia. The authors used a cross-sectional study design via the 2019 Ethiopian Demographic and Health Survey. For our study, we used 3837 births recorded by mothers in seven regions of Ethiopia. In this study, the author employed the Bayesian and classical logistic regression models. The study found that the household size, number of under-five children, Sex of child, twin, births in the last five years, and breastfeeding status are significant predictors of child mortality in Ethiopia. Consequently, governmental, non-governmental, and other concerned bodies should focus on targeted healthcare interventions for mothers and children by updating their health intervention policies. In addition, improved health services are needed for better health care for children and mothers. Education should be given to mothers during pregnancy and after birth. This helps improve health for mothers and children, along with addressing other risk factors.

## Introduction

Child death is the proportion of live births in a given year that die before the child reaches the age of five years. Annually, millions of children less than five years of age die^1^. The burden of child mortality is unevenly distributed, with a disproportionately high burden in southern Asia and sub-Saharan Africa. For example, South Asia and sub-Saharan Africa account for more than 80% of all under-five deaths. Six nations, including Ethiopia, account for 50% of all under-five deaths worldwide^2–4^. Ethiopia has endorsed the Sustainable Development Goals (SDGs), specifically SDG 3.2, which seeks to reduce under-five mortality to lower than 25 per 1,000 live births by 2030^5^.

The Ethiopian Ministry of Health began planning by 2035 to reduce child death rates to less than 30 mortalities per 1000 live births^6^. Getting to this low point will necessitate progress in the population’s socioeconomic position and improvements in the direct facilities offered by the health sector^7^. Furthermore, the sustainable development goal aims for under-five deaths per thousand live births. The global under-five death rate fell by 59% between 1990 and 2018, from 93 deaths per 1000 live births to 39 in 2018. Despite this significant improvement, ensuring the existence of children remains a top priority. Every day in 2018, approximately 15,000 children under five died, an unacceptably large number of entirely avoidable child mortality^8,9^, the termination of the Millennium Development Goal, the worldwide community decided on a new agenda, the strategic development goals, to eliminate preventable deaths from fresh burns and under-five-year-old children. The goal is to cover all nations and reduce child deaths to no more than 25 child deaths for every thousand live births.

The 120 member States have met the targets of strategic development goals for child death, and 21 more are predicted to meet the goal by 2030^10^. Child mortality is a key indicator of a nation’s child health development since it reflects the economic, environmental, and social situations in which children live, especially their healthcare^11^. Approximately 5.9 million children died worldwide in 2015. According to the World Health Organization report (WHO), the likelihood of death of children by the age of five is highest in African countries, which have 81 per thousand live births, over seven times greater than in European countries, which have 11 per thousand live births. Fewer developed nations reported 76 fatalities, which is 11 times greater than the number reported in high-income countries^12^.

Africa, especially sub-Saharan Africa, is still facing serious issues. As the region with the highest child death rate in the world, 98 fatalities per 1,000 live births occurred in 2012. Sub-Saharan Africa is home to all 16 nations with under-five death rates of more than 100 fatalities per 1,000 live births^13^. While Ethiopia has achieved improvements in child healthcare over the last five years, the child mortality rate has decreased from 123 − 88 fatalities per 1,000 live births. The mortality rate remains unacceptably high, with one in 11 children dying before reaching the age of five^14^.

Furthermore, some regions in Ethiopia have a high number of child deaths. For example, according to the 2011 EDHS, Afar has the highest number of under-five deaths per 1000 live births^15^. In these Ethiopian regions, socioeconomic and environmental factors influence child health, including family income, access to health, and parental education. The effects of child death are evaluated via biological, socioeconomic, behavioral, and environmental factors^16^.

Research has shown that low levels of maternal education, unsafe drinking water and sanitation, low family income, a short birth interval, a short breastfeeding time, and the place of delivery are among the determinants of under-five mortality^9,17–22^. Even as Ethiopia strides in lowering under-five mortality rates by enhancing access, quality, and utilization of skilled care, essential newborn care, and management of preterm and low birth weight, the rates remain alarmingly high in some regions, especially the Afar, Somalia, and Benishangul Gumuz regions^23^. Hence, this study explored the factors contributing to under-five mortality in these high-risk regions of Ethiopia.

Understanding the complexities of child mortality and its influencing factors is crucial for effective policy formulation and targeted interventions. This research endeavors to contribute valuable insights into the realization of child health, supporting efforts to achieve the SDGs and promoting the well-being of children worldwide. In light of this gap, the main objective of this study was to investigate the impact of demographic, socioeconomic, and environmental determinants on child mortality and to identify risk factors through Bayesian logistic regression, utilizing a non-informative prior distribution and aiming to gain insight for effective decision-making by healthcare professionals, policymakers, and communities toward making appropriate interventions to reduce under-five mortality.

## Methods and materials

### Data source

The study used the Ethiopian Demographic Health Survey (EMDHS). The data are a cross-sectional and nationally representative survey that measures key indicators for countries to collect data to inform their policies and practices. It follows a consistent sampling and data collection protocol, coding, and questionnaires, and follows the results to be assessed in different countries. Participants in the study were selected via a two-stage stratified sampling method based on DHS data protocols that ensure national representation. The 2019 EMDHS for women aged 15–49 years consists of 3,837 households, which aligns with the specific objectives of our study and includes only those with complete information on maternal health, child health, and other related variables.

Our analysis focused on mothers who gave birth within the five years of the study. Since the first five years of the postnatal period (postpartum) is a crucial period for child and maternal health. During this period, mothers experience ongoing psychological and physiological adjustments, including the establishment of breastfeeding, recovery from childbirth, and adaptation to new caregiving roles. These factors can significantly influence both the child’s development and the mother’s health^24^. Additionally, this period reflects current child and maternal health policy national priorities.

Access to the raw data is available on the DHS website upon the authors’ reasonable request. It adheres to strict confidentiality and ethical standards. All DHS datasets are fully anonymized before being released to researchers. In particular, all personally identifiable information (PII), including names, household identifiers, and addresses are removed. Moreover, informed consent and ethical approval were obtained from the DHS at the time of data collection on the basis of the national and international ethical standards. For our study, we used the publicly available dataset alone after securing approval from the DHS Program. The data are used only for academic purposes by their data use agreement. The forms for appealing access to raw data are available on the website (www.dhsprogram.com). The authors have requested the ICF International’s Review Board. Finally, the authors received an authorization letter from the ICF.

### Study design and study population

This research used a cross-sectional study design based on 2019 EMDHS data. The target population covers all mothers who gave birth to a live baby or infant in their lifetime in the selected seven regions of Ethiopia.

### Study variables

The response variable of this research is the proportion of the number of child deaths per mother for her lifetime. For this analysis, the key predictors of child mortality were drawn on the basis of the literature on child mortality and morbidity in the least developed countries. The authors classified these factors as socioeconomic, environmental, and demographic factors. The independent variables are presented in Table 1.

**Table 1:** Descriptions of the demographic, socioeconomic, and environmental variables.

Variable	Code	Description
Child is alive	Child-status	0 = No, 1 = Yes
Age of mother	Agecat	1. 15-19, 2. 20–24, 3. 25-29, 4. 30–34, 5. 35–39, 6. 40–44, 7. 45–49
Age	Age	Number of years
Region	Region	1. Afar, 2. Oromia, 3. Somali, 4. Benishangul, 5. Snnpr, 6. Gambela, 7. Harari
Residence	Residence	1 = urban & 2 = rural
Mother education	Edulevel	(1) No education. (2) primary and above
Toilet	Toilet	(1) No toilet facility. (2) toilet facility access
NumhhsMe	hhsmembers	Number of household members
Under 5 children	NoUnder5	Number of under 5 children in the household
Sex of household head	sexhhshead	1 = male & 2 = female
Age of household head	agehhshead	Number of years
Literacy	Literacy	(1) Cannot read at all. (2) able to read
Cooking fuel	Cooking	(1) Electricity/kerosene/charcoal/other. (2) Wood/grass/animal dung/other
Wealth Index	Wealth	1 = Poor, 2 = middle, 3 = rich
Total children ever born	Totchild	Number of children
Births in last five years	Last5ybirths	Number of live births
Age of mother at 1st birth	Age1stbirth	Number of years
Currently pregnant	currpreg	1 = No or unsure& 2 = yes
Current contraceptive	currconmeth	1. Not using. 2 pill/iud/injection/diaphram/other
Current use	currusmeth	(1) No method. (2) modern/traditional methods
Contraceptive use	cousi	(1) Modern/traditional methods. (2) does not intend to use
Birth order	Order	Number of birth order
Child is twin	Childtwin	(1) Single birth. (2) multiple
Sex of child	sexchild	1 = Male & 2 = female
Current age of child in months	cuagechild	Number of months
Duration of breastfeeding	durbreast	(1) Ever breastfed, not currently breastfeeding. (2) never breastfed
Place of delivery	placedel	1. Home. 2 health facility
Delivery cesarean	decrease	0 = No & 1 = Yes
Birth history	indexnum	Index number
Ever attended school	everscho	0 = No & 1 = Yes

### Methods

This study used binary outcome models to examine the factors associated with child death in seven regions of Ethiopia. The analysis was carried out with R statistical software version 4.4.3: https://projects.r-project.org and with STATA statistical software version 17: https://www.stata.com. On the basis of previous studies, Bayesian inference may produce a more accurate estimate in the presence of prior information than the classical likelihood estimation procedure does^25^, whereas many researchers often opt for non-informative priors when employing the Bayesian approach^26^. The author enhanced the model’s accuracy and improved parameter value estimation by incorporating non-informative priors derived from historical data. As a result, Bayesian analysis yields more robust and reliable results than traditional classical inference methods do^27^. Despite various research efforts in child mortality, there is a noticeable research gap concerning the application of Bayesian inference to explore child mortality in Ethiopia. This study used classical and Bayesian logistic regression models to examine the associations between selected variables under five years of age and child mortality.

### Frequentist logistic regression model

The general form of a logistic regression model can be written as

1 $$\:\text{log}\left[\text{Pr}\left({y}_{i}|X,\beta\:\right)\right]=\text{log}\left(p\right)=\text{log}\left(\frac{p}{1-p}\right)\:\:\:\:\:\:\:\:\:\:\:\:\:\:\:\:\:\:\:\:\:\:\:\:\:\:\:\:\:\:\:\:\:\:\:\:\:\:\:\:\:\:\:\:\:\:\:\:\:\:\:\:\:\:\:\:\:$$

2 $$\:\:\:\:\:\:\:\:\:\:\:\:\:\:\:\:\:\:\:\:\:\:\:\:\:\:\:\:\:\:\:\:\:\:\:\:\:\:\:\:\:\:\:\:\:\:\:\:\:\:\:\:\:\:\:\:\:\:\:\:\:={\beta\:}_{0\:}+\:{\beta\:}_{1}{X}_{1}\:+{\beta\:}_{2}{X}_{2}+\dots\:.+{\beta\:}_{\text{p}}{X}_{\text{p}}\:\:\:\:\:\:\:\:\:\:\:\:\:\:\:\:\:\:\:\:\:\:\:\:\:\:\:\:\:\:\:\:\:\:\:\:\:\:\:$$

where $\:{X}_{1}$, *· · ·*,$\:{X}_{p}$ are the factors of child mortality; $\:{\beta\:}_{1}$, *· · ·*, $\:{\beta\:}_{p}$ are the regression coefficients; and $\:{\beta\:}_{0}$ is the intercept. The coefficient of regressions represents the direction and magnitude of the effects of the X design matrix of the factors on the binary response variable$\:{\:y}_{i}$. *β* is a vector of the coefficients of the regression. *p* in Eq. (1) denotes the probability that a child has, and $\:\left(\frac{p}{1-p}\right)\:$ represents the odds of child mortality among those children who are exposed to the factors compared with those who are not exposed to the factors. This suggests that *β* represents the log odds ratio of death among children who are exposed to the predictors relative to those who are not exposed to the predictors.

### Bayesian logistic regression model

The Bayesian approach to the logistic model follows the standard procedure for all Bayesian analyses. This involves defining the likelihood function for the data and the prior distribution for the parameters in the proposed regression model. The data likelihood function is subsequently multiplied by the prior distribution for the parameter estimates to obtain the posterior density. The probability of death or success among children differs from one child to another, depending on their risk factors. Hence, the likelihood function for the child mortality data follows a binomial distribution. Let y = ($\:{y}_{1},{y}_{2}$,…, $\:{y}_{n}$) be n independent binomial or binary random variables with the probability mass function defined as

3 $$\:\text{Pr}\left({y}_{i}|{\theta\:}_{i}\right)=\left(\frac{{m}_{i}}{{y}_{i}}\right){\theta\:}_{i}^{{y}_{i}}{\left(1-\theta\:\right)}^{{m}_{i-{y}_{i}}},{y}_{i}=\text{0,1},\dots\:,{m}_{i},{m}_{i}>1,\:i=\text{1,2},3,\dots\:,n.\:\:\:\:\:\:\:\:\:\:\:\:$$

where $\:{\theta\:}_{1}$= *F*(X′*β*), *F* is a cumulative distribution function. *X*′ is a *p*-dimensional covariate. *β* is the vector of unknown regression coefficients. $\:{m}_{i}$ is the number of observations for the $\:{i}^{th}$ child. The logit link function is obtained by setting

4 $$\:{F}^{-1}\left({\theta\:}_{I}\right)=\text{log}\left(\frac{{\theta\:}_{i}}{1-{\theta\:}_{i}}\right)={\text{X}}^{{\prime\:}}\beta\:\:\:\:\:\:\:\:\:\:\:\:\:\:\:\:\:\:\:\:\:\:\:\:\:\:\:\:\:\:\:\:\:\:\:\:\:\:\:\:\:\:\:\:\:\:\:\:\:\:\:\:\:\:\:\:\:\:\:\:\:\:\:\:\:\:\:\:\:\:\:\:\:\:\:\:\:\:\:\:\:\:\:\:\:\:\:\:\:\:\:\:\:\:\:\:$$

From Eq. (3), the likelihood function of binomial data is defined as

5 $$\:\text{f}\left(\text{y},{\upbeta\:}\right)=\left(\frac{{m}_{i}}{{y}_{i}}\right){\theta\:}_{i}^{{y}_{i}}{\left(1-\theta\:\right)}^{{m}_{i-{y}_{i}}}\:\:\:\:\:\:\:\:\:\:\:\:\:\:\:\:\:\:\:\:\:\:\:\:\:\:\:\:\:\:\:\:\:\:\:\:\:\:\:\:\:\:\:\:\:\:\:\:\:\:\:\:\:\:\:\:\:\:\:\:\:\:\:\:\:\:\:\:\:\:\:\:\:\:\:\:\:\:\:\:\:\:\:\:\:\:\:\:\:\:$$

By using Eq. (4), we can show that

6 $$\:{\theta\:}_{i}=\frac{\text{e}\text{x}\text{p}({\text{X}}^{{\prime\:}}\beta\:={\beta\:}_{0\:}+\:{\beta\:}_{1}{X}_{1\text{i}}\:+{\beta\:}_{2}{X}_{2\text{i}}+\dots\:.+{\beta\:}_{\text{p}}{X}_{\text{p}\text{i}})}{1+\text{e}\text{x}\text{p}({\text{X}}^{{\prime\:}}\beta\:={\beta\:}_{0\:}+\:{\beta\:}_{1}{X}_{1\text{i}}\:+{\beta\:}_{2}{X}_{2\text{i}}+\dots\:.+{\beta\:}_{\text{p}}{X}_{\text{p}\text{i}})}\:\:\:\:\:\:\:\:\:\:\:\:\:\:\:\:\:\:\:\:\:\:\:\:\:\:\:\:\:\:\:\:\:\:\:\:\:\:\:\:\:\:$$

From expression (5), the likelihood contribution from the $\:{i}^{th}$ child is written as

7 $$\:\text{f}\left(\text{y},{\upbeta\:}\right)=\left(\frac{{m}_{i}}{{y}_{i}}\right){\left(\frac{\text{e}\text{x}\text{p}\left({{X}_{i}}^{{\prime\:}}\beta\:\right)}{1+\text{e}\text{x}\text{p}\left({{X}_{i}}^{{\prime\:}}\beta\:\right)}\right)}_{i}^{{y}_{i}}{\left(\frac{1}{1+\text{e}\text{x}\text{p}\left({{X}_{i}}^{{\prime\:}}\beta\:\right)}\right)}^{{m}_{i-{y}_{i}}}\:\:\:\:\:\:\:\:\:\:\:\:\:\:\:\:\:\:\:\:\:\:\:\:\:\:\:\:\:\:\:\:\:\:\:\:\:\:\:\:\:\:\:\:\:$$

The unknown parameters in expression (6) are $\:{\beta\:}_{0\:}$, $\:{\beta\:}_{1}$, …, and $\:{\beta\:}_{\text{p}}$. Therefore, we need to specify a prior distribution for these unknowns. We used the most common priors for logistic regression parameters, which are of the form $\:{\beta\:}_{\text{j}}$ ∼N ($\:{\mu\:}_{\text{j}}$,$\:\:{\beta\:\sigma\:}_{j}^{2}$). The distribution of the parameter estimates can be expressed explicitly as

8 $$\:\text{f}\left({\beta\:}_{j}\right)=\frac{1}{\sqrt{2\pi\:{\sigma\:}_{j}}}\text{e}\text{x}\text{p}\left[-\frac{1}{2}{\left(\frac{{\beta\:}_{j}-{\mu\:}_{j}}{{\sigma\:}_{j}}\right)}^{2}\right]\:\:\:\:\:\:\:\:\:\:\:\:\:\:\:\:\:\:\:\:\:\:\:\:\:\:\:\:\:\:\:\:\:\:\:\:\:\:\:\:\:\:\:\:\:\:\:\:\:\:\:\:\:\:\:\:\:\:\:\:\:\:\:\:\:\:\:\:\:\:\:\:\:\:\:\:\:\:\:\:\:\:\:\:\:\:\:\:\:\:\:\:\:\:\:\:\:\:$$

The most common choice for $\:{\mu\:}_{j}$ is zero, and $\:{\sigma\:}_{j}$ is usually chosen to be large enough to be considered noninformative. As stated earlier, the posterior distribution is obtained by multiplying the prior distribution over all the parameters by the full likelihood function as

9 $$\:\text{f}\left({\upbeta\:}|{y}_{i},{x}_{{ip}}\right)=\left({y}_{i}|\beta\:\right)\times\:f\left(\beta\:\right)\:\:\:\:\:\:\:\:\:\:\:\:\:\:\:\:\:\:\:\:\:\:\:\:\:\:\:\:\:\:\:\:\:\:\:\:\:\:\:\:\:\:\:\:\:\:\:\:\:\:\:\:\:\:\:\:\:\:\:\:\:\:\:\:\:\:\:\:\:\:\:\:\:\:\:\:\:\:\:\:\:\:\:\:\:\:\:\:\:\:\:\:\:\:\:\:\:\:\:\:\:\:\:$$

It follows from expression (9) that

10 $$\:\text{f}\left({\upbeta\:}|{x}_{ip}\right)=\prod\:_{i=1}^{n}\left(\frac{{m}_{i}}{{y}_{i}}\right){\left(\frac{\text{exp}\left({{X}_{i}}^{{\prime\:}}\beta\:\right)}{1+\text{exp}\left({{X}_{i}}^{{\prime\:}}\beta\:\right)}\right)}_{i}^{{y}_{i}}{\left(\frac{1}{1+\text{exp}\left({{X}_{i}}^{{\prime\:}}\beta\:\right)}\right)}^{{m}_{i-{y}_{i}}}\:\:\times\:\:\:\:\prod\:_{j=1}^{p}\frac{1}{\sqrt{2\pi\:{\sigma\:}_{j}}}\text{e}\text{x}\text{p}\left[-\frac{1}{2}{\left(\frac{{\beta\:}_{j}-{\mu\:}_{j}}{{\sigma\:}_{j}}\right)}^{2}\right]\:\:\:\:\:\:\:\:\:\:\:\:\:\:\:\:\:\:\:\:\:\:\:\:\:\:\:\:\:\:\:\:\:\:\:\:\:\:\:\:\:\:\:\:\:\:\:\:\:\:\:\:\:\:\:\:\:\:\:$$

The above expression (10) has no closed-form expression. In this study, we use the metropolis-Hastings algorithm with the Bayes package in R to simulate parameter estimates from the posterior distributions. Note that these parameter estimates are subject to Monte Carlo error, which is difficult to quantify. Therefore, the authors have chosen a very long run in which convergence was reached at 4000 after a burn-in period of 1000 and thinning of every 99th element of the chain for each model.

## Results

### Descriptive analysis

As shown in Table 2, among the 3837 children, 7% died. Most mothers reside in rural areas (77.8%). About 57.2% of mothers do not have formal education. This may impact mothers’ educational opportunities and socioeconomic status for their children. A significant number of mothers (42.1%) use unprotected water sources. This may cause waterborne diseases and serious health risks.

This finding highlights the need for better access to safe drinking water and improved water infrastructure. Almost 50% of mothers do not have access to a suitable toilet facility. This may have major implications for hygiene, public health, and environmental sanitation. This indicates a need for public health interventions and infrastructure improvements to provide adequate sanitation facilities.

Additionally, 66.3% of mothers were illiterate. This shows a major challenge in access to learning and education opportunities. A high illiterate rate has a wide-ranging impact on the overall quality of life of children. A significant number of mothers (95%) use traditional cooking fuel. These fuels are less expensive and more readily available but may cause high health risks for child mortality.

A majority of mothers (58.5%) were poor. This may cause a high prevalence of child mortality. A significant number of mothers had never breastfed (13.5%). This indicates a need for education support programs to encourage breastfeeding. The majority of mothers (74.7%) were not using any current contraceptive methods. A total of 53.7% of mothers delivered their homes. This may reflect economic constraints, accessible issues, and cultural preferences.

**Table 2:** Demographic and socioeconomic characteristics for categorical variables.

Characteristics	Measures	Number	Percent
Child status	Alive	3569	93
	Not alive	269	7
Age category	15–19	232	6.0
	20–24	825	21.5
	25–29	1243	32.4
	30–34	786	20.5
	35–39	452	11.8
	40–44	222	5.8
	45–49	77	2.0
Region	Afar	652	17.0
	Oromia	719	18.7
	Somali	637	16.6
	Benishangul	530	13.8
	Gambela	450	11.7
	Harari	447	11.6
	Dire dawa	402	10.5
Place of residence	Urban	850	22.2
	Rural	2987	77.8
Mother education	No education	2195	57.2
	Primary and above	1642	42.8
Source of drinking water	Unprotected	1615	42.1
	Protected	2222	57.9
Toilet facility	No toilet facility	1830	47.7
	Toilet facility access	2007	52.3
Religion	Orthodox	1011	26.3
	Protestant	697	18.2
	Muslim	2055	53.6
	Other	74	1.9
Sex of household head	Male	2941	76.6
	Female	896	23.4
Sex of child	Male	1997	52.0
	Female	1840	48.0
Cooking fuel	Elec./Kero/cha/other	201	5.2
	Wood/grass/animal dung/other	3636	94.8
Currently pregnant	Ever or not currently breastfeeding	3315	86.4
	Never breastfed	522	13.6
Current contraceptive method	No method	2865	74.7
	Modern method/traditional methods	972	25.3
Child is twin	Single birth	3715	96.8
	Multiple	122	3.2
Duration of breastfeeding	Ever not currently breastfeeding	2213	57.7
	Never breastfeed	1624	42.3
Place of delivery	Home	2061	53.7
	Health facility	1776	46.3
Delivery by cesarean section	No	3658	95.3
	Yes	179	4.7

Table 3 shows that the mean age of mothers is 28 years, with minimum and maximum ages of 15 and 49 years, respectively. The mean age of the children was 2.5 years, with minimum and maximum ages of 0 and 4.92 years, respectively.

**Table 3:** Demographic and socioeconomic characteristics for continuous variables.

Covariate	Number	Minimum	Maximum	Mean	Std deviation
Current age	3837	15	49	28.20	6.508
Number of household	3837	1	24	6.37	2.565
Number of under 5children	3837	0	5	1.85	0.893
Age of household head	3837	15	98	37.36	13.255
Total children ever born	3837	1	15	4.24	2.564
Births in last five years	3837	1	5	1.81	0.748
Age of mother at 1st birth	3837	10	37	18.25	3.956
Birth order	3837	1	15	3.84	2.515
Age of child in months	3837	0	59	29.19	17.155
Birth history	3837	1	5	1.40	0.613

Table 4 shows that mothers aged greater than 45 years have a higher prevalence of child mortality than young mothers. This result coincides with other findings, which stated that old maternal age is linked to increased risks in child health. During Pregnancy, biological factors, hypertension, declining reproductive health, gestational diabetes impacting child survival. Low birth weights in offspring have a greater likelihood of chromosomal abnormalities. This are related to higher child and infant mortality. In middle and low-income countries, old mothers can face limited access to quality healthcare. Mainly in underserved areas their age can lead to reproduce cumulative socioeconomic difficulties and limited education. This contributes to worse child survival outcomes^28,29^.

Among the seven regions, Somali (89), Benishangul (85), and Gambela (78) have high rates of child mortality. However, there is no statistically significant evidence to suggest that these regions have highly significant differences in under-five child mortality. Compared with female children, male children have a high prevalence rate of mortality (79).

Compared with single-born children, twin-born children have a very high prevalence of child mortality (303). Research findings in Ethiopia have consistently revealed that twin-born children have a greater risk of child mortality due to prematurity and low birth weight^30^.

Mothers who deliver by cesarean section have a higher prevalence rate of child mortality (95) than mothers who do not use (69). But, there is no statistical evidence to suggest that mothers who deliver by cesarean section have a higher prevalence of child mortality than those who do not.

**Table 4:** Prevalence of Under-five mortality in Ethiopia, 2019, *N* = 3837.

Variables	Category	Total	Number of died	Mortality rate	*P* value
Child status	Died	3,568	269	75	-
Age groups	15–19	232	22	95	0.000
	20–24	825	61	74	
	25–29	1,243	72	58	
	30–34	786	46	59	
	35–39	452	35	77	
	40–44	222	12	54	
	45–49	77	21	273	
Region	Afar	652	35	54	0.274
	Oromia	719	41	57	
	Somali	637	57	89	
	Benishangul	530	45	85	
	Gambela	450	35	78	
	Harari	447	29	65	
	Dire dawa	402	27	67	
Place of residence	Urban	850	60	71	0.261
	Rural	2,987	209	70	
Mother education	No	2,195	151	69	0.385
	Primary	1,642	118	72	
Source of drinking water	Unprotected	1,615	109	67	0.260
	Protected	2,222	160	72	
Toilet facility	No	1,830	123	67	0.290
	Toiltfaciaccess	2,007	146	73	
Religion	Orthodox	1,011	73	72	0.490
	Protestant	697	56	80	
	Muslim	2,055	135	66	
	Other	74	5	68	
Sex of hhds head	Male	2,941	217	74	0.007
	Female	896	52	58	
Sex of child	Male	1,997	157	79	0.008
	Female	1,840	112	61	
Cooking fuel	Electric	201	10	50	0.582
	Wood	3,636	259	71	
Wealth index	Poor	2,239	161	72	0.591
	Middle	438	35	80	
	Rich	1,160	73	63	
Currently pregnant	Ever not now	3,315	226	68	0.237
	Neverbreastfed	522	43	82	
Current contraceptive method	No method	2,865	209	73	0.218
	Modern/tradition	972	60	62	
The child is twin	Single birth	3,715	232	62	0.000
	Multiple	122	37	303	
Duration of breastfeeding	Ever not now	2,213	152	69	0.012
	Never breastfed	1,624	117	72	
Place of delivery	Home	2,061	158	77	0.11
	Health facility	1,776	111	63	
Delivery by cesarean session	No	3,658	252	69	0.51
	Yes	179	17	95	

### Frequentist logistic regression analysis

The results from the frequentist logistic regression are summarized in Table 5. Table 5 shows that significant predictors of child death include the number of household members, the number of under five children, sex of the child’s head, the total number of children ever born, the number of births in the last five years, the number of twins children, sex of the child, the current age of the child, age of the household head, and the breastfeeding status. Some factors, such as access to toilet facilities, place of delivery, and cesarean section delivery, do not have a statistically significant effect (significant codes: 0 ‘***’ 0.001 ‘**’0.01 ‘*’ 0.05 ‘.’ 0.1 ‘ ’ 1.

**Fig. 1:**
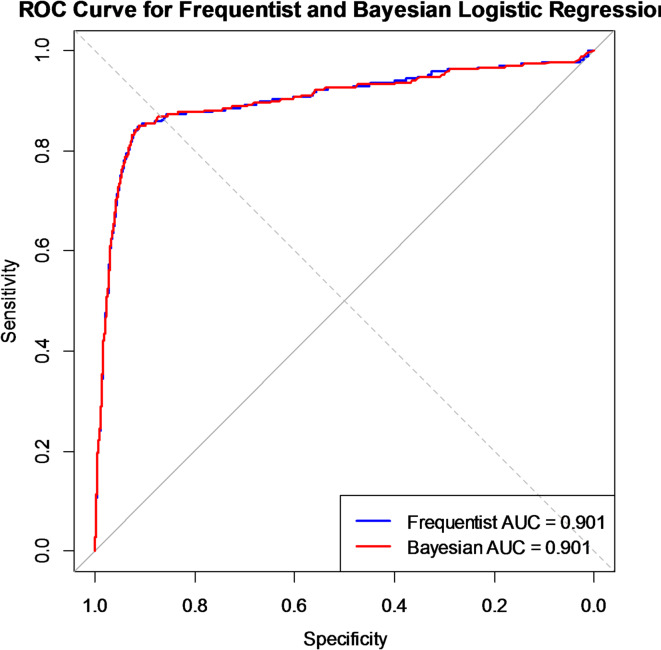
The ROC curves for the frequentist and Bayesian logistic regression models.

As illustrated in Fig. 1, each point on the curve has a different cutoff value. The position of that point is displayed as the sensitivity (true positive rate) at that cutoff value on the Y axis. 1 specificity (false positive rate) at that cutoff value on the x-axis. The ROC curve graphically depicts the tradeoff between maximizing the true positive rate and minimizing the false positive rate. The AUC score (0.9) indicates that the models performed well, with extremely high accuracy in differentiating between the two classes. This level of performance suggests that the models are very trustworthy and effective for practical application in forecasting outcomes.

**Table 5:** Standard logistic regression model.

Coefficients	Estimate	Std. error	z value	Pr(>|z|)
Intercept	-1.79	0.57	3.14	0.002**
toilt facility access	0.18	0.17	1.06	0.290
numhme	-0.13	0.05	-2.49	0.013*
under5	-2.21	0.13	-17.16	0.000***
sexchildhmale	0.55	0.20	2.70	0.007 **
agehhead	-0.02	0.01	-2.79	0.006 **
totalch	0.19	0.04	4.44	0.000 ***
birthlast5y	1.61	0.13	12.74	0.000***
childtwinsingle birth	-1.20	0.32	3.78	0.000 ***
sexchildmale	0.42	0.16	2.67	0.008 **
curragechild	0.01	0.01	1.98	0.048 *
durbreastfeed	0.47	0.19	2.50	0.012*
placedelihome	0.28	0.18	1.58	0.11
delicayes	0.23	0.35	0.66	0.51

### Bayesian logistic regression analysis

Figure 1 reveals that both models have strong predictive power and perform well. The identical AUC values and overlapping ROC curves suggest that both models offer nearly identical classification accuracies for this dataset. An AUC value close to 1 reflects a high discriminating ability between the positive and negative classes. This indicates that the models perform at a strong level of classification accuracy. In this case, since the performance of the two approaches is similar, the choice between the frequentist and Bayesian methods may not significantly affect model accuracy. However, other factors, such as model interpretation ability, computational efficiency, and the ability to incorporate prior knowledge can influence the decision. The Bayesian method offer advantages in uncertainty quantification, and providing posterior distributions for parameters, which is valuable in many contexts. While the 95% Bayesian credible interval and the 95% frequentist confidence interval offer a comparable range, their interpretations differ owing to the philosophical differences between these approaches. The Bayesian framework allows the incorporation of prior knowledge by using prior distributions and provides posterior distributions, offering more information than a simple point estimate does. Thus, the decision to use one model over the other depends on factors beyond mere classification performance, such as the need for uncertainty quantification, interpretation ability and leveraging prior knowledge. The fit diagnostics suggest that the Bayesian logistic regression model provides stable and consistent predictions. This indicates good model calibration and predictive performance.

The diagnostic outputs of the Bayesian model suggest that all the parameters have effectively converged. The diagnostics include several key indicators to confirm the reliability and accuracy of the posterior estimates. Convergence is assessed via the Rhat statistic, which is the potential scale reduction factor. For all the parameters in this model, the Rhat values are consistently 1.0, which indicates strong convergence across the MCMC chains. Rhat values close to 1 suggest that the chains have successfully mixed. The variance between chains is similar to the variance within chains. This is a critical check to ensure that the posterior distribution has been explored sufficiently by the MCMC algorithm.

The Monte Carlo standard error (MCSE) values are small, indicating precise estimates. The MCSE quantifies the error due to the finite number of MCMC samples, reflecting the accuracy of the parameter estimates. Smaller MCSE values indicate that the estimates are likely to be close to the true values, as they reduce the noise or uncertainty introduced by the sampling process.

An effective sample size (neff) measures the equivalent number of independent samples from the posterior distribution. Large neff values indicate better mixing of the chains and more reliable parameter estimates. In this case, all the parameters have substantial neff values, reinforcing the credibility and robustness of the estimates drawn from the MCMC procedure. A high neff suggests that the samples represent the posterior distribution, with minimal autocorrelation. Additionally, the Pareto k values, which stem from the leave-one-out cross-validation (LOO-CV) diagnostic, are all less than 0.7. A lower Pareto k values below 0.7 indicate that no individual observation exerts excessive influence on the model’s predictions. This confirms the stability and representation of the model when unseen data are being predicted. This implies that no observation is highly affected by the overall fit.

Overall, these diagnostics confirm that the Bayesian model performs reliably in terms of fit and predictive power. The combinations of well-behaved Rhat, small MCSE, substantial neff, and acceptable Pareto k values confidently indicate that the model has converged properly. The estimates of the Bayesian models are precise and reliable. Hence, the predictions are not unduly influenced by any single data point. Thus, the diagnostic results support the conclusion that the model is well-suited for inference. This can be justified for further use in decision-making and additional analyses.

**Fig. 2:**
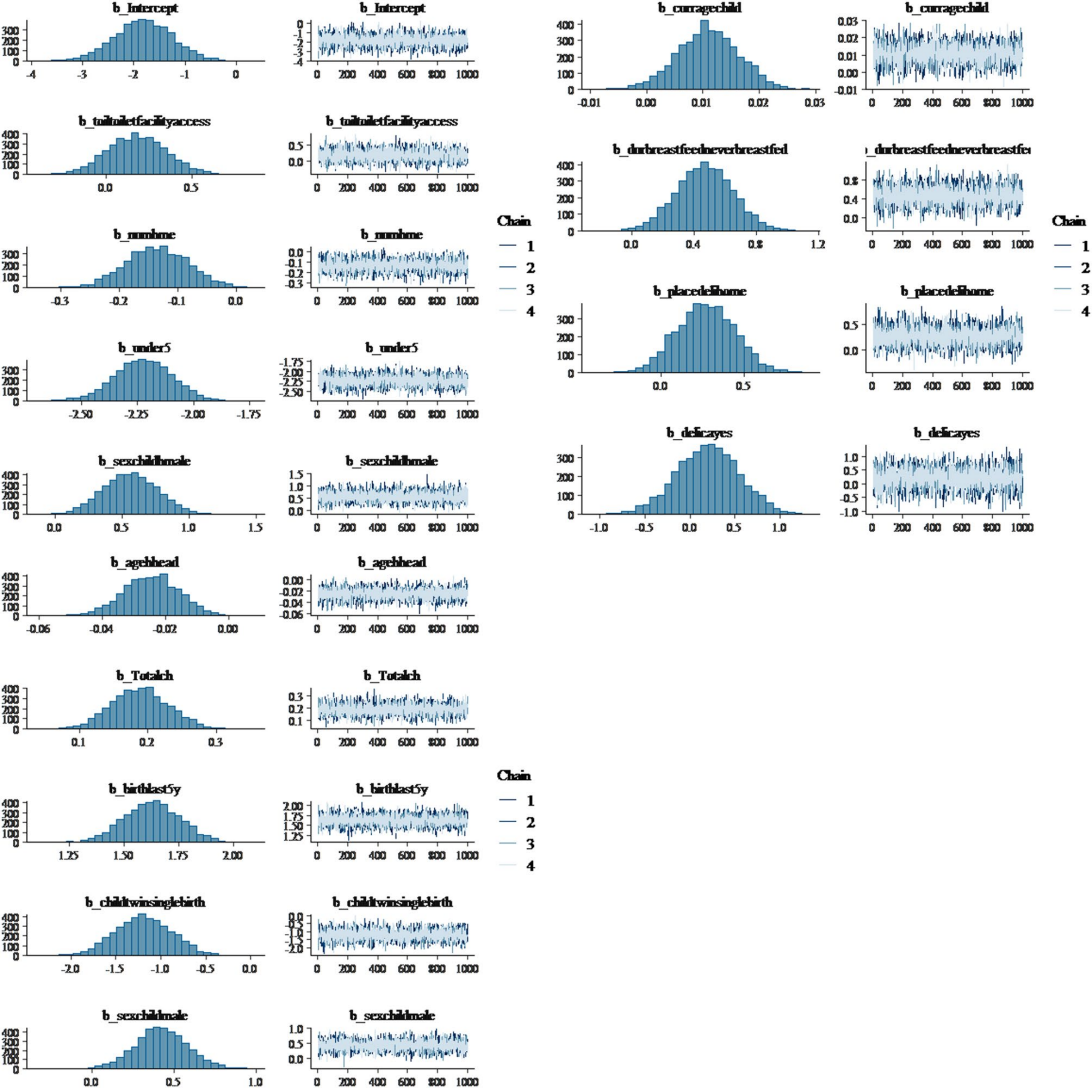
Posterior distribution and trace plot for Bayesian logistic model parameters.

In Fig. 2, trace plots from MCMC sampling are typically used in Bayesian statistical analysis to visualize the convergence and behavior of the chains for different parameters. In the Multiple Chains, each plot displays four chains represented by different colors: blue, green, red, and purple, indicating that four separate Markov chain Monte Carlo (MCMC) runs were performed for each parameter. Each subplot corresponds to a different model parameter, labeled at the top of each plot, such as Intercept, electric facility and so on. The horizontal axis denotes the iteration number of the MCMC sampling process. Typically, as the number of iterations increases; the chains should converge to the posterior distribution. The vertical axis indicates the value of the parameter at each iteration. Generally, the trace plots show the evolution of parameter estimates over iterations for multiple chains.

The density map displayed in Fig. 2 highlights the importance of posterior predictive plots as valuable tools for validating models in Bayesian analysis. The authors evaluated the model’s suitability and predictive capacity by comparing the observed data with the model’s posterior distribution. The alignment between the observed and anticipated data indicates a credible model. Plotting actual data against predicted data points helps assess the model’s accuracy.

**Fig. 3:**
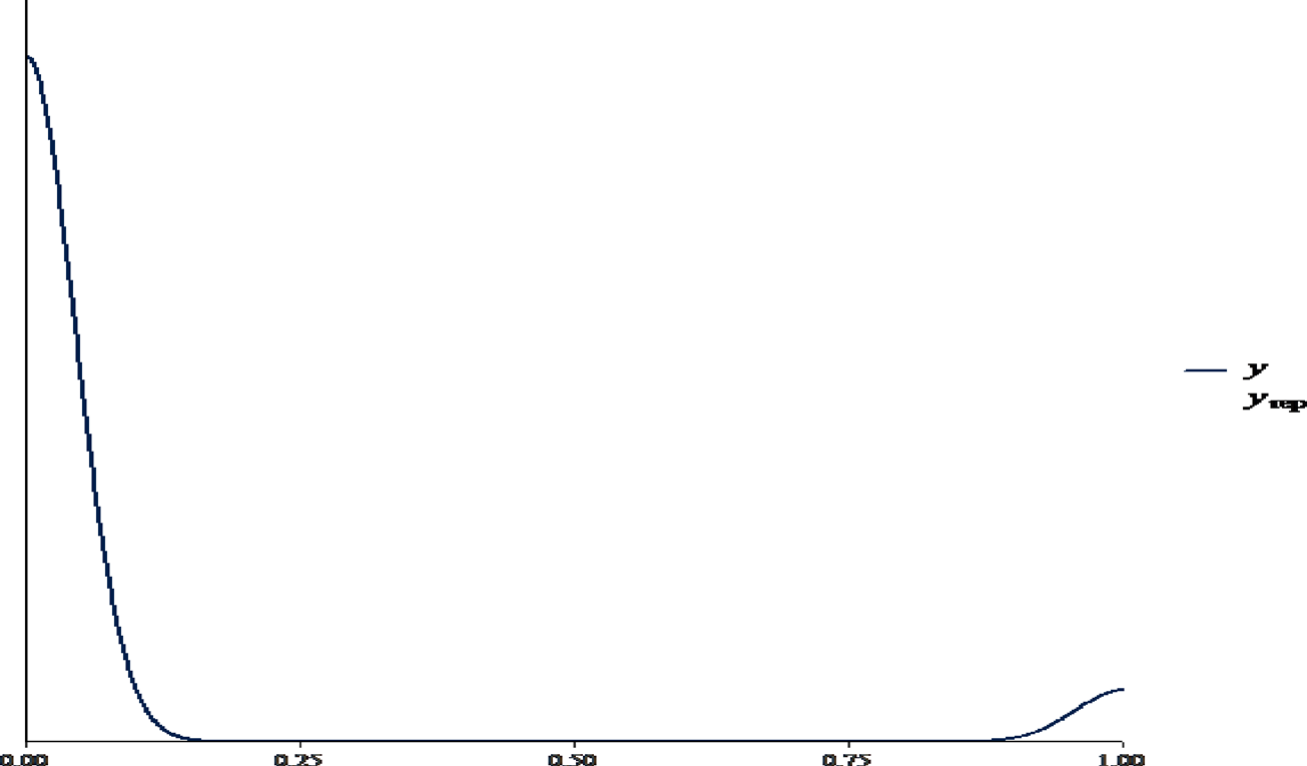
Bayesian Posterior Predictive Plot.

In Fig. 3, the Bayesian posterior predictive plot was utilized to evaluate the model’s alignment with the observed data and the predicted data with the actual observations. The x-axis denotes the probability of child mortality. The y-axis shows the observed and predicted probabilities. The lines in the plot exhibit similar patterns, with a high density near 0, indicating that most of the data fall within the lower probability range. Moving rightward along the x-axis, there is a noticeable decline in density. The lines appear to closely overlap across most of the distribution, particularly in the left part of the graph. Toward the upper end of the x-axis (near 1.00), there is a slight divergence where the predicted data exhibit a small upward bump such that the observed data do not meet the mirror. However, the overall fit seems quite good, as the observed and predicted densities are mostly aligned.

**Fig. 4:**
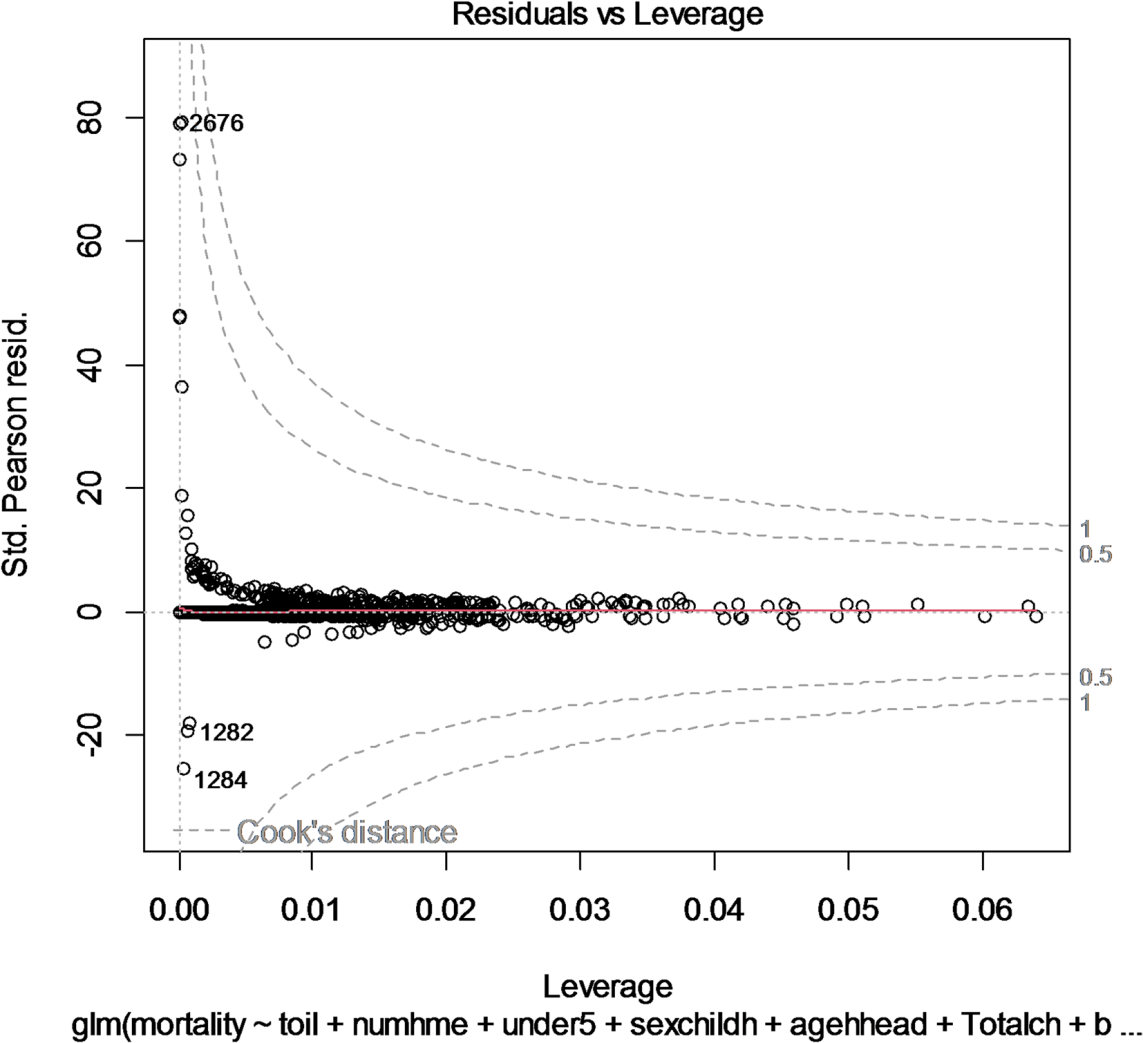
Residual vs. leverage plot.

Figure 4 shows the spread of standardized residuals changes when the leverage, sensitivity of the fit to a change, increases. This can also be used to check heteroscedasticity and nonlinearity. The dotted red line represents Cook’s distance, and spots outside the dotted line have a significant influence. The y-axis shows the residuals, which are the discrepancies between the observed values and the values predicted by the regression line.

The writing in the top left corner of the graph indicates that the residuals are plotted on the y-axis and the leverage is plotted on the x-axis. There appears to be no evident relationship between residuals and leverage. This shows that the residuals are independent of leverage. There are several data points with high leverage values. These points may be influential outliers and should be investigated further. Overall, the graph indicates that the regression analysis could be reliable.

In Table 6, the intercept estimate is -1.81, suggesting that all the variables are zero, the log odds of child mortality are negative coefficients. The 95% confidence interval clearly shows that the true value falls between − 2.91 and − 0.68. This result providing strong evidence that the intercept is negative. The coefficient for toilet facility access is 0.18, suggesting that having access to toilet facilities increases the logits of the outcome. However, the lower bound of the 95% confidence interval (-0.15, 0.53) slightly overlaps zero. It indicates weaker certainty regarding the positive effect. The estimate for the number of household members is -0.14, implying that as the number of household members increases, the log odds of the outcome decrease. The 95% CI between (-0.24 and − 0.03) does not include zero, signifying a significant negative association.

The coefficient of the number of children under five is -2.23, indicating a strong negative association and significant decreases in the logits of the outcome with CI (-2.48, -1.97). The positive coefficient (0.56) suggests that male children have higher log odds of child mortality than female children, with a credible interval (0.15, 0.98). The coefficient of the age of the household is small. But, a credible interval (-0.04, -0.01) significantly suggests that older household heads slightly decrease the log odds. The 95% confidence interval for the total number of children was between 0.11 and 0.27. This finding indicates that there is significant evidence of a positive effect on child mortality.

The estimate for births in the last five years is 1.63, suggesting that having had a birth in the last 5 years increases the log odds of child mortality with the 95% CI (1.39–1.88). The effect of being a twin or single birth is positive, with the credible interval (-1.81, -0.58) indicating statistically significant effect. The estimate for the sex of the child is 0.42, indicating a positive association with the outcome, with a 95% credible interval (0.11 to 0.73). The estimate of the duration of breastfeeding is 0.47, indicating that not breastfeeding is associated with higher log odds of the outcome within the 95% credible interval of 0.09 and 0.84. The estimate for the place of delivery is 0.28, suggesting a positive association with the outcome for home delivery. However, the 95% confidence intervals indicate uncertainty about the significance of this effect. The Rhat estimate of 1.00 shows that all the parameters have converged. Additionally, the higher values of the Bulk ESS and Tail ESS estimates indicate desirable effective sample sizes, ensuring accurate estimation of the posterior distributions.

**Table 6:** Bayesian logistic regression estimates.

Regression coefficients	Estimate	Est. error	l-95% CI	u-95% CI	Rhat	Bulk ESS	Tail ESS
Intercept	-1.81	0.56	-2.91	-0.68	1.00	4,728	3,394
toiltfacilityaccess	0.18	0.17	-0.15	0.53	0.00	4,375	2,875
numhme	-0.14	0.05	-0.24	-0.03	1.00	3,302	2,623
under5	-2.23	0.13	-2.48	-1.97	1.00	3,320	3,278
sexchildhmale	0.56	0.20	0.15	0.98	1.00	4,499	3,033
agehhead	-0.02	0.01	-0.04	-0.01	1.00	4,173	2,743
Totalch	0.19	0.04	0.11	0.27	1.00	3,961	2,882
birthlast5y	1.63	0.12	1.39	1.88	0.00	3,688	2,953
childtwinsinglebirth	-1.19	0.31	-1.81	-0.58	1.00	51.00	2,885
sexchildmale	0.43	0.16	0.11	0.73	1.00	4,919	2,535
curragechild	0.01	0.01	0.00	0.02	1.00	4,870	3,200
durbreastfeed	0.47	0.19	0.09	0.84	1.00	4,075	3,224
placedelihome	0.28	0.17	-0.05	0.61	1.00	3,855	3,181
delicayes	0.21	0.34	-0.49	0.86	1.00	4,700	2,987

In Table 7, the intercept represents the baseline odds of the outcome when all the predictors are zero. The credible interval is relatively narrow. The 95% confidence interval indicates that the odds are significantly less than 1, suggesting modest uncertainty. Households with access to toilet facilities increase the odds of child mortality by 20% compared with those without access. But, the wide interval (0.86, 1.68) suggests that the effect is not significant. For an additional household member, the odds of the outcome decrease by approximately 13%, which is supported by the 95% CI [0.79, 0.97], indicating a significant effect with a negative value. A mother having a child less than 5 years of age significantly reduces the odds of the outcome by 89%; the 95% CI (0.08, 0.14) confirmed a strong significant effect with a negative value. The odds of having male children are 75% greater than female children, with a relatively wide credible interval (1.17, 2.66).The number of total children in the household slightly increases the odds of child mortality by 21% with 95% CI (1.11, 1.31). Mothers who have given birth in the last 5 years significantly increase the odds of child mortality by 409% with the 95% CI [3.98, 6.54]. This indicates that there is a strong, significant positive effect.

A child being a single birth significantly decreases the odds of child mortality by 70%, with a credible interval (0.16, 0.55). The odds of male children are 54% greater than female children, with a 95% CI [1.12, 2.12]. The odds of children who were never breastfed are 60% greater than the odds of children who were breastfed, with a 95% CI (1.10, 2.32). The odds of delivery at home are 32% greater than the odds of delivery at a health center with the 95% CI (0.94, 1.86).

**Table 7:** Posterior predicted model estimate.

Variable	Estimate	Est. error	Q2.5	Q97.5
Intercept	0.16	1.75	0.05	0.51
toiltfacilityaccess	1.20	1.19	0.86	1.70
numhme	0.87	1.05	0.79	0.97
under5	0.11	1.14	0.08	0.14
sexchildhmale	1.75	1.22	1.16	2.66
agehhead	0.98	1.01	0.96	0.99
Totalch	1.21	1.04	1.12	1.31
birthlast5y	5.10	1.13	4.01	6.55
childtwinsinglebirth	0.30	1.36	0.16	0.56
sexchildmale	1.54	1.17	1.12	2.08
curragechild	1.01	1.01	1.00	1.02
durbreastfeed	1.60	1.21	1.09	2.32
placedelihome	1.32	1.19	0.95	1.84
delicayes	1.23	1.40	0.61	2.36

## Discussions

Predicting child mortality requires models that are generalized across diverse populations and settings. Informative priors are useful in some cases that can be rigid by imposing assumptions that may not hold across different contexts. Alternatively, Noninformative priors, offer greater flexibility when we estimate complex predictors such as demographic, healthcare access, and socioeconomic factors^31^.

In logistic regression, noninformative priors allow the model to rely more heavily on the data, minimizing the risk of bias from subjective prior information. This makes the analysis more objective and data driven, providing more insight into the relationship between predictors and child mortality. Noninformative priors also serve as a form of regularization, reducing the likelihood of overfitting without imposing strong constraints on the model parameters. Furthermore, it provides a neutral baseline for sensitivity analyses, offering a cleaner interpretation of the model’s results than informative priors. This approach enhances the model’s ability to generalize across different datasets and populations. Thus, noninformative priors are more robust tool for understanding and predicting child mortality^32^.

The frequentist confidence interval specifies a range within which the true parameter value is expected to fall with a certain level of confidence on the basis of the sample data, whereas, the Bayesian confidence interval specifies the actual probability that the true parameter lies within the specified range, given the observed data and the prior beliefs. The authors also note that these interpretational differences can influence policy and clinical decisions. A Bayesian credible interval can provide more informative and additional information in clinical or policy settings, which is often more pertinent in real-world settings^32^.

The prior for the regression coefficients is specified via a normal distribution, with a mean of 0 and a standard deviation of 2.5. This normal distribution reflects our initial beliefs about the likely values of the coefficients before observing any data. A mean of 0 suggests that a priori, we believe the coefficients are close to zero. The standard deviation or scale parameter controls how much uncertainty we have about the coefficients. A large standard deviation indicates greater uncertainty in the coefficient values. Conversely, a smaller standard deviation would signal more confidence in the belief that the coefficients are near zero. In Bayesian analysis, validating these prior assumptions is critical for ensuring that the priors align with the observed data. Therefore, the results of the prior predictive checks, sensitivity analysis, posterior predictive checks, and LOO and WAIC criteria ensure that our priors are reasonable, well calibrated and improve the updated model’s predictive power^33^.

The AIC is used to assess the performance of the frequentist model. Whereas the IOOIC and WAIC used to evaluate the performance of the Bayesian model. The AIC value is lower than the IOOIC and WAIC values. This indicates that the model with the lowest AIC is marginally superior. Nonetheless, the differences are minimal, suggesting that the models perform comparably^34^.

The analysis highlights that both the frequentist and Bayesian logistic regression models offer nearly identical classification accuracies. An AUC close to 1 signifies a strong ability to distinguish between positive and negative classes; reinforcing the robust performance of both models in this particular dataset. The similarity in classification accuracy of the two models suggests that performance alone may not drive the choice between the two models. Instead, practical considerations such as uncertainty quantification, computational resources, and the ability to incorporate prior knowledge are more influential in selecting the appropriate approach for specific contexts. Bayesian methods, interpretative power through posterior distributions offer advantages in scenarios requiring more nuanced insights into uncertainty and model parameter behavior^32,35^.

In this study mothers whose ages are greater than 45 years have a higher prevalence rate of child mortality than young mothers. Among the seven regions, Somalia, Benishangul, and Gambela have high prevalence rates of child mortality. The descriptive results show that male children have a higher prevalence rate of mortality than female children. Mothers who use wood, grass, and animal dung have a high prevalence rate of child mortality than mothers, who use electricity, kerosene, and charcoal,

Twin-born children have a very high prevalence rate of child mortality compared with single-born children. Mothers whose deliveries by cesarean section have a high prevalence rate of child mortality compared with mothers who do not undergo cesarean sections^36,37^.

This finding revealed that family size has a significant effect on under-five child mortality in both rural and urban areas. This finding coincides with other studies showing that household size and economic status are associated with child mortality. Households with 6 or more children have a greater rate of child mortality than those with 1–2 and 3–5 children^38–40^.

This study shows that male children have an increased risk of death compared to female children. This study’s findings coincide with other studies^41,42^. Therefore, the results suggest that specific interventions targeting gender issues are needed to reduce the risk of child mortality. Children who were not breastfed have significantly greater risk of under-five child mortality than breastfed. This finding coincides with other studies^43–45^ and shows that the positive impact of breastfeeding on child mortality is well documented. This result showed that new births in the last five years are a significant factor in under-five-child mortality. This study coincided with other findings^46,47^, which consistently showed that firstborn children face a greater risk of death within the first five years than other children. The substantial influence of a recent birth within the last 5 years could be compared to studies on maternal health and child spacing, which often highlight the impact of short birth intervals on health outcomes.

In the Bayesian results, the intercept with a large standard deviation indicated a wide variation in the baseline level of the outcome variable. This finding coincided with other studies^48,49^, showing significant variability in the intercept and reflecting diverse baseline conditions in different population’s settings. In this study, the effect of toilet facility is negligible with high variability. Many studies emphasize the importance of sanitation facilities in improving the health status of children. In this study, the number of children under five years of age had a mean effect with notable variability. This result coincides with^50^, showing that having young children is often linked to increased healthcare needs, a higher risk of illness, and child mortality.

The research findings show that the age of the household head significantly affects child mortality with minimal variability. These results coincide with other studies that often bring stability and experience, potentially benefiting outcomes. But, the effects are usually small and context-dependent^51,52^.

In this study, twin-born children had a greater risk of child mortality than single-born children did. This finding coincided with^53^. These findings show that children with multiple births have a higher mortality rate than single births. The study revealed that female-headed households have less experience with child mortality than male-headed households. This study revealed that the current age of the child has a substantial effect on child mortality with minimum variability. The results provide valuable insights into the determinants of under-five child mortality with several significant predictors. Comparing these findings with those in the literature can enhance understanding and highlight areas for future research. The intercept has a large standard deviation, indicating substantial variability in the baseline level of the outcome across different regions. This suggests that baseline conditions vary widely among different population’s settings^54–56^.

Access to toilet facilities shows a negligible mean effect but with considerable variability. This highlights the importance of contextual factors in determining the impact of sanitation on health outcomes. The number of household members has a negligible effect on the outcome. This suggests a balance between the potential positive and negative impacts of household size, such as increased support versus resource competition. Having children under 5 years of age has no average effect but has notable variability. This reflects differing health and resource dynamics in households with young children, which can lead to varied outcomes. The sex of the child has some effects, with male children having a slight positive effect on under-five child mortality. The age of the household head has a negligible mean effect with minimal variability, indicating that this factor does not significantly influence child mortality^57^.

The total number of children in the household has a negligible mean effect with some variability. This suggests that the impact of having more children on the outcome is context-dependent, balancing resource constraints and social support. Birth within the last 5 years has a negligible mean effect, with considerable variability. This finding indicated that the impact of recent can vary widely. Never breastfeeding has a small positive effect with high variability. This finding coincides with^58^ and suggested that lack of breastfeeding can depend on alternative nutrition and health care practice.

## Conclusions

Most regions in Ethiopia have higher child mortality rates. This is due to socioeconomic challenges and poor access to health services. The government must prioritize initiatives in regions with high child mortality rates. Focus on enhancing health education campaigns, allocating more resources, and improving infrastructure.

Older mothers face a higher risk of losing a child. So, improved antenatal care outreach initiatives should be implemented for mothers 45 and older. These initiatives should involve delivery and careful monitoring during pregnancy and after childbirth.

Twin-born children are related to a higher risk of child mortality. Specialized follow-up services for twin birth delivery should be provided. Children who were never breastfed had a greater risk of mortality. Media and health professionals should promote breastfeeding education in the first six months. Male children have a greater chance of under-five mortality. To address gender-based health disparities, community-based awareness initiatives should be adopted. Current births in the last five years have increased the risk of child mortality. Community-based family planning health facilities must adopt.

Families with large newborns and household sizes have a greater risk of child mortality. Female-headed households have reduced child mortality rates due to better child healthcare practices. Social and economic support programs for female-headed households should be set up so they can access child healthcare services.

Most significant variability suggested that their impacts are context dependent. This can depend on local health practices, socioeconomic conditions, and cultural differences. In general, the authors suggested that more detailed research in specific contexts must be implemented. The government should support large families through nutrition supplements, child health grants, and healthcare subsidies, and improve sanitation. Thus, policymakers and practitioners should take interventions by considering these contextual factors for better child healthcare practices.

### Limitations of the study

The Ethiopian Demographic and Health Survey rely on cross-sectional data. Sampling variability, missing data, and social desirability bias will affect the data. Despite its limitations, the DHS is a reliable source of national data. It provides valuable and useful data related to child mortality in Ethiopia.

## Data Availability

The datasets analyzed in this study are available on the DHS website (www.dhsprogram.com) upon reasonable request and approval.

## Abbreviations

EDHS: Ethiopia Demographic Health Survey
MOH: Ministry of Health
GTPE: Growth Transformation Plan of Ethiopia
MDGs: Millennium Development Goals
FDRMST: Federal Democratic Republic of Ministry of Science and Technology
MCMC: Markov Chain Monte Carlo
LOOIC: Leave one out information criteria
AIC: Akaike Information Criterion
WAIC: Watanabe-Akaike Information Criterion
